# Chitosan oligosaccharide inhibits skull resorption induced by lipopolysaccharides in mice

**DOI:** 10.1186/s12903-019-0946-7

**Published:** 2019-11-27

**Authors:** Ke Guo, Zong Lin Liu, Wen Chao Wang, Wei Feng Xu, Shi Qi Yu, Shan Yong Zhang

**Affiliations:** 1 Department of Oral Surgery, Shanghai Ninth People’s Hospital, College of Stomatology, Shanghai Jiao Tong University School of Medicine; Shanghai Key Laboratory of Stomatology, 639 ZhiZaoJu Road, Shanghai, 200011 China; 2 Shanghai Ninth People’s Hospital, School of Biomedical Engineering, Shanghai Jiao Tong University, Shanghai, China

**Keywords:** Chitosan oligosaccharide, LPS, Micro-CT, TRAP staining, Bone resorption

## Abstract

**Background:**

Low-molecular-weight chitosan oligosaccharide (LMCOS), a chitosan degradation product, is water-soluble and easily absorbable, rendering it a popular biomaterial to study. However, its effect on bone remodelling remains unknown. Therefore, we evaluated the effect of LMCOS on lipopolysaccharide (LPS)-induced bone resorption in mice.

**Methods:**

Six-week-old male C57BL/6 mice (n = five per group) were randomly divided into five groups: PBS, LPS, LPS + 0.005% LMCOS, LPS + 0.05% LMCOS, and LPS + 0.5% LMCOS. Then, the corresponding reagents (300 μL) were injected into the skull of the mice. To induce bone resorption, LPS was administered at 10 mg/kg per injection. The mice were injected three times a week with PBS alone or LPS without or with LMCOS and sacrificed 2 weeks later. The skull was removed for micro-computed tomography, haematoxylin-eosin staining, and tartrate-resistant acid phosphatase staining. The area of bone damage and osteoclast formation were evaluated and recorded.

**Results:**

LMCOS treatment during LPS-induced skull resorption led to a notable reduction in the area of bone destruction; we observed a dose-dependent decrease in the area of bone destruction and number of osteoclasts with increasing LMCOS concentration.

**Conclusions:**

Our findings showed that LMCOS could inhibit skull bone damage induced by LPS in mice, further research to investigate its therapeutic potential for treating osteolytic diseases is required.

## Background

Chitosan oligosaccharide (COS) is a product of chitosan degradation. Chitin is the second largest biological macromolecule polysaccharide in nature, and chitosan is the product of chitin deacetylation. Chitosan is a non-toxic biocompatible molecule that possesses antibacterial properties and promotes the growth of osteoblasts, cell proliferation, and differentiation [1–4]. Chitosan is an integral component of a new type of mixed biofilm particularly pertaining to the field of guided tissue regeneration (GTR) and guided bone regeneration (GBR) [5, 6]. In the field of bone-defect scaffold materials, a chitosan composite porous scaffold was shown to have better porosity and osteogenic activity with higher bone formation volume and rate than the conventional bone scaffold material, making it a promising new material for repairing bone defects [7]. Although the use of chitosan in GBR, GTR, and bone defect scaffold applications has been widely reported, it has certain disadvantages, such as its large molecular weight, water insolubility, low degradation rate, and relatively slow absorption rate. In contrast, COS, which is the product of chitosan degradation, has a low molecular weight and is water-soluble and easily absorbed, rendering it a popular biomaterial to study in recent years.

COS has been widely used in agriculture, industry, biomedical biomaterials, food bioengineering, and other fields for several decades. Several studies have also suggested COS inhibits apoptosis and promotes the healing of bone defects [8–11]. COS is also reported to promote the proliferation and differentiation of osteoblasts, and the expression of genes related to bone defects [12, 13]. Nevertheless, the effect of low-molecular-weight chitosan oligosaccharide (LMCOS) on bone remodelling has not been reported. In this study, we evaluated the effect of LMCOS on inflammatory bone destruction induced by lipopolysaccharide (LPS) in mice. We established an inflammatory bone destruction model and treated the mice with different concentrations of LMCOS.

## Methods

### Materials and equipment

Six-week-old male C57BL/6 mice were from Shanghai West Poole-Baykay Laboratory Animal Co., Ltd. (Shanghai, China); bacterial LPS and a tartrate-resistant acid phosphatase (TRAP) detection kit were purchased from Sigma-Aldrich (St. Louis, MO, USA); chitosan oligosaccharide powder (polymerization degree, 2–10; average molecular weight, 1000 Da; purity, > 98%; deacetylation degree, 99%). The micro-CT scanner (μCT-100) used in this study was purchased from Scanco Medical AG, Brüttisellen, Switzerland. This study was approved by the Ethics Committee of Shanghai Ninth People’s Hospital (SH9H-2019-A502–1).

### Inflammatory bone destruction model and LMCOS treatment

LPS was dissolved in phosphate-buffered saline (PBS) at a concentration of 1 g/L. The LPS bone destruction model was described previously; briefly, bone destruction was induced by injecting LPS between the subcutaneous tissue and bone periosteum of the head in mice [14]. The mice were randomly divided into five groups (n = five per group): control mice injected with PBS only (300 μL); mice injected with LPS (200 μL) and PBS (100 μL); mice injected with LPS (200 μL) and 0.005% LMCOS (100 μL); mice injected with LPS (200 μL) and 0.05% LMCOS (100 μL); mice injected with LPS (200 μL) and 0.5% LMCOS (100 μL). The mice were injected three times per week and euthanized 2 weeks later. To reduce pain for euthanasia, the mice were briefly anaesthetized in an isoflurane-filled box to induce early unconsciousness, and then the mice were killed with cervical dislocation.

### Micro-computed tomography

The cranium of each mouse was harvested, and the soft tissue around the bone was separated for micro-computed tomography (micro-CT). The scanning parameters were 70 kV, 114 mA, and a scanning thickness of 50 μm. A bone mass analysis was performed to evaluate bone damage quantitatively after three-dimensional reconstruction.

### Haematoxylin and eosin (H&E) staining

The calvarial bone tissue of each mouse was fixed in 4% paraformaldehyde for 24 h and then washed overnight. Subsequently, the tissue was decalcified with 10% EDTA at 4 °C, dehydrated by a graded series of ethanol, and embedded in paraffin after n-butyl alcohol exchanges. Each specimen was sectioned to a near-far-median sagittal section thickness of 4 μm. Following sectioning, H&E staining was performed to visualize cranial cap bone defects under a microscope.

### TRAP staining

The TRAP stain was prepared according to the manufacturer’s instructions. Calvarial bone specimens were deparaffinized with dimethylbenzene and processed with a graded series of ethanol concentrations. The TRAP stain was then applied to the tissues and incubated at 37 °C for 1 h. Subsequently, the tissues were re-stained with haematoxylin for 2 min, cleared with xylene, and mounted and sealed with neutral gum. The tissue slides were observed under an optical microscope, and TRAP-positive cells (brown) were counted and subjected to statistical analysis.

### Statistical analysis

The data are presented as the mean ± standard deviation, and statistical analysis was performed using the SPSS 21.0 software package (IBM, Armonk, NY, USA). A single factor analysis of variance and Student-Newman-Keuls (SNK) test were used for the statistical analysis. A *P-*value < 0.05 indicated a significant difference between groups.

## Results

### LMCOS decreases the formation of bone resorption pits induced by LPS

**Fig. 1:**
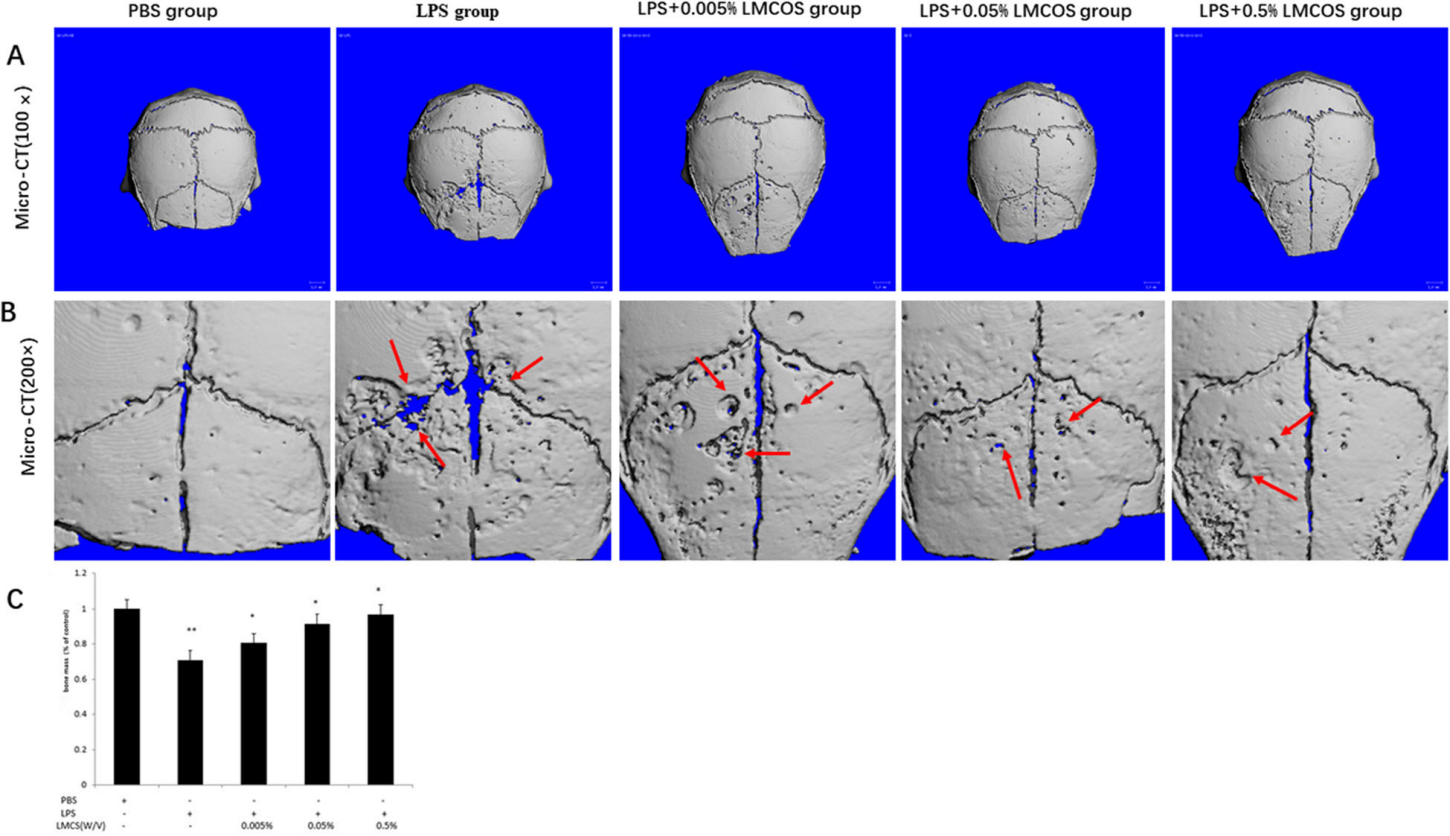
**a** and **b**. Mouse calvarial bone resorption was detected with Micro-CT. PBS group; LPS group; LPS + 0.005% LMCOS group; LPS + 0.05% LMCOS group; LPS + 0.5% LMCOS group; **c**. Calvarial bone volume was measured by micro-CT, and calvarial bone mass measurement was expressed by the bone mass ratio between the experimental group and the control group (**p < 0.05, *p < 0.05). The arrows represented bone resorption pits

The formation of cranial resorption pits was assessed using micro-CT. Compared with the PBS-only control group, the formation of cranial resorption pits was more apparent in the LPS group (Fig. 1a and b). However, LPS-induced bone resorption pits were significantly reduced in mice treated with LMCOS compared with the LPS treated group, and the bone resorption pits were reduced further with increasing LMCOS concentrations (Fig. 1a and b).

The statistical analysis of the micro-CT scans of the skull caps of mice showed that the skull bone mass of mice in the LPS group was reduced compared with that of mice in the blank control group. The bone mass of the LPS + 0.005% LMCOS group, LPS + 0.05% LMCOS group, and LPS + 0.5% LMCOS group was higher than that of the LPS group. Furthermore, the number of bone trabeculae was higher in the high-dose group than in the low-dose group. Compared with the LMCOS group, the PBS group and LPS group exhibited statistically significant differences. At the same time, bone indexes such as bone resorption cavities, bone trabeculae thickness, and number showed that the high-concentration LMCOS group had stronger inhibiting effects on bone resorption than that of the low concentration LMCOS group (Fig. 1c) (**p < 0.05, *p < 0.05).

### LMCOS limits LPS-induced cranial bone damage

**Fig. 2:**
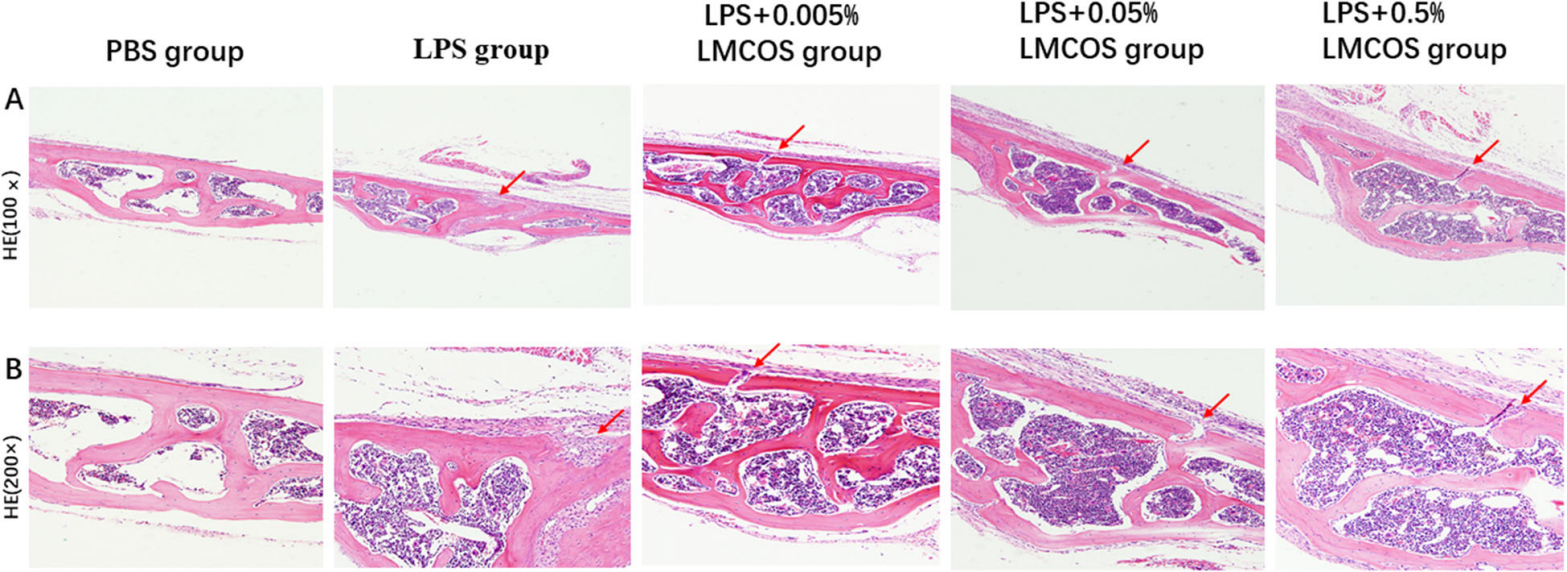
**a** and **b**. Mouse calvarial bone resorption was examined with H-E staining. PBS group; LPS group; LPS + 0.005% LMCOS group; LPS + 0.05% LMCOS group; LPS + 0.5% LMCOS group. The arrows represented bone resorption pits

The H&E staining results showed that the cranium of LPS-treated mice was notably damaged compared with that of mice in the PBS control group (Fig. 2a and b). However, in mice treated with LMCOS, the cranial damage induced by LPS was significantly inhibited, and bone resorption was significantly reduced. The bone damage was further decreased by increasing concentrations of LMCOS (Fig. 2a and b).

### LMCOS reduces the number of TRAP-positive cells

**Fig. 3:**
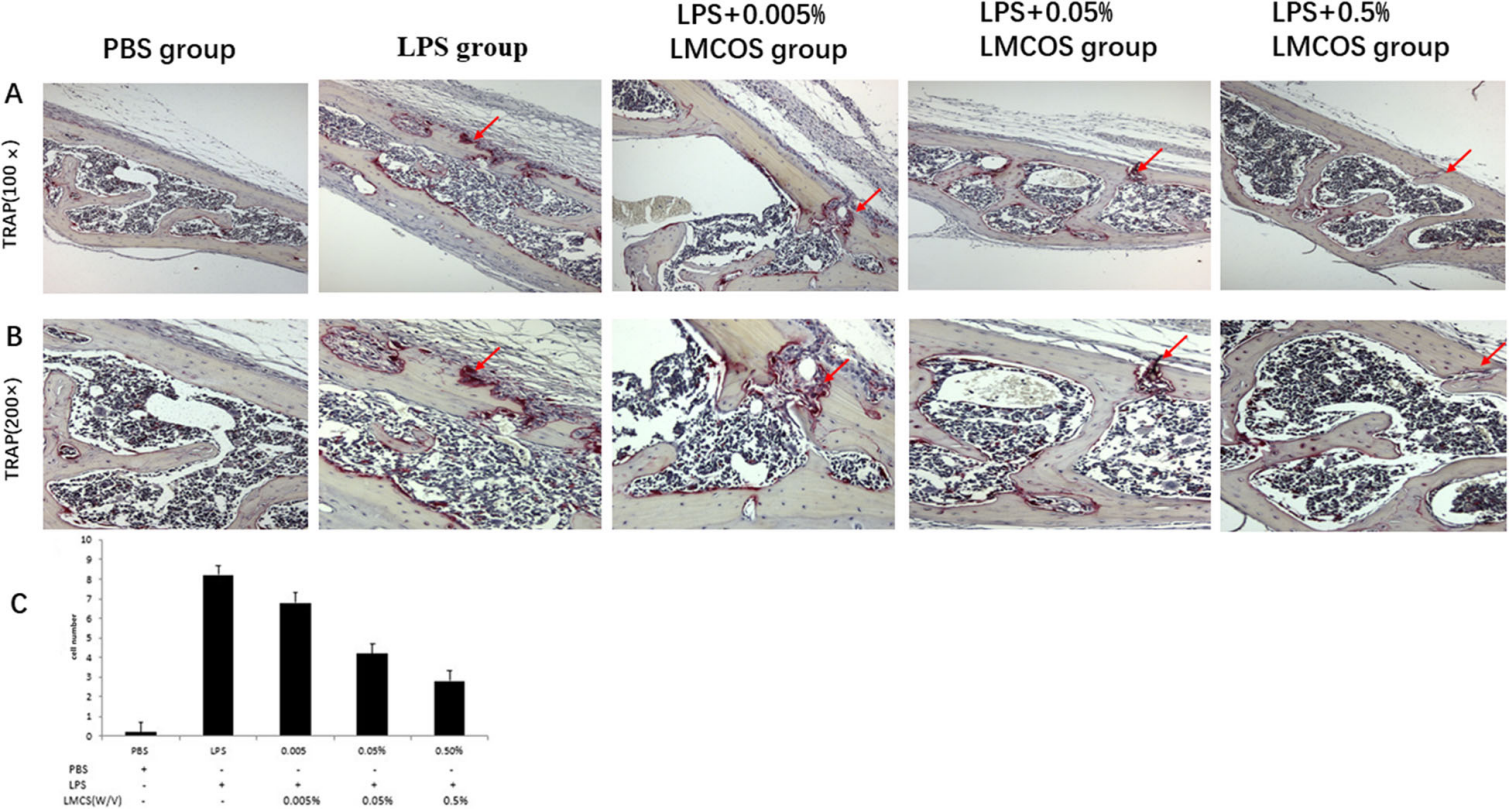
**a** and **b**. Mouse calvarial bone resorption was examined with TRAP staining. PBS group; LPS group; LPS + 0.005% LMCOS group; LPS + 0.05% LMCOS group; LPS + 0.5% LMCOS group; **c**. Quantitative analysis of TRAP-positive cells (**p < 0.05, *p < 0.05). The arrows represented bone resorption pits (TRAP, × 10)

The TRAP staining showed that LMCOS inhibited osteoclastogenesis induced by LPS (Fig. 3a and b). The number of TRAP-positive cells was graphed and subjected to statistical analysis (Fig. 3c). The number of osteoclasts increased in the LPS group compared with the control group. At the same time, the number of osteoclasts in the LPS + 0.005% LMCOS group, LPS + 0.05% LMCOS group, and LPS + 0.5% LMCOS group was lower than that in the LPS group alone. The number of osteoclasts in the high-dose group was less than that in the low-dose group. Compared with the LMCOS group, the PBS group and LPS group exhibited statistically significant differences. In the processing of osteoclasts, the group with a high concentration of LMCOS exhibited stronger inhibitory effects on bone resorption than that of the group with a low concentration of LMCOS. LMCOS treatment significantly reduced the number of TRAP-positive cells induced by LPS.

## Discussion

In GBR, GTR, and related technologies, bone healing materials remain the most critical components of the technologies. Currently, the main clinical applications of GBR and GTR include autogenous bone [15], heterogeneous bone [16], allogeneic bone [17–19], and artificial synthetic materials [20]. The ideal bone healing materials should possess the following characteristics: 1) biocompatibility and non-toxicity; 2) biodegradability and absorbency; 3) a biological activity that simulates the structure of the bone matrix and promotes the regeneration of bone tissue [21].

In recent years, numerous studies have shown that chitin, chitosan, COS, and their derivatives also have certain effects on in vitro cultured cells, mainly to promote cell proliferation and differentiation. The osteogenic properties of these materials in bone defect reconstruction have also been evaluated in several studies [22, 23]. A new chitosan composite porous scaffold has been studied and entered the application stage. Chitosan-composite porous stent, a new material for repairing bone defects, has better porosity and good osteogenic activity, with bone formation volume and bone formation rate superior to those of conventional bone scaffold materials [24]. However, its large molecular weight, water insolubility, low degradation rate, and relatively slow absorption rate have restricted its applications in medicine. As a degradation product of chitosan, LMCOS has the advantages of low molecular weight, biocompatibility, biodegradability, antibacterial properties, and absorbability [25]. Compared with chitosan, LMCOS has an improved degradation rate and solubility. Furthermore, studies have shown that LMCOS can promote osteoblast proliferation [4]. Nevertheless, the role of LMCOS in inhibiting osteoclasts is yet to be determined.

Results of our preliminary study provided early evidence of the LMCOS effect on osteoclasts, indicating its inhibitory effect on the osteoclast process. To further explore the ability of LMCOS to inhibit the osteoclast process, we utilized a mouse LPS-induced cranial bone destruction model to evaluate the effect of LMCOS on inflammatory bone damage [26]. LPS induces mononuclear macrophages to secrete a variety of inflammatory mediators and promote the fusion of osteoclast precursors, maintains mature osteoclast activity, and stimulates osteoclasts to perform bone resorption functions [27]. We tested different concentrations of LMCOS in an experimental animal model to assess its dose-dependent inhibition of the osteoclast process. Using micro-CT scanning, we verified that LPS induced significant cranial bone resorption; however, LMCOS was able to reduce bone resorption lacunae in a dose-dependent manner. Results of the H&E staining also showed that LMCOS could significantly inhibit bone damage induced by LPS. TRAP staining further confirmed that the number of osteoclasts was decreased with LMCOS intervention compared with that in mice injected with LPS only. Therefore, our findings indicated that LMCOS limits LPS-induced bone destruction by inhibiting osteoclast formation.

## Conclusions

In summary, our study demonstrated the ability of LMCOS to inhibit skull bone destruction induced by LPS in mice. Our findings suggested the therapeutic application of LMCOS for treating bone diseases, although the specific molecular mechanism needs further studies.

## Data Availability

The dataset used and/or analyzed during the current study are available from the corresponding author on reasonable request.

## Abbreviations

COS: Chitosan oligosaccharide
GBR: Guided bone regeneration
GTR: Guided tissue regeneration
LMCOS: Low-molecular-weight chitosan oligosaccharide
LPS: Lipopolysaccharide
micro-CT: micro-computed tomography
PBS: Phosphate-buffered saline
TRAP: Tartrate-resistant acid phosphatase
